# Race for a COVID-19 vaccine

**DOI:** 10.1016/j.ebiom.2020.102817

**Published:** 2020-05-22

The entire world is united in facing the emergency of the coronavirus disease 2019 (COVID-19) pandemic: we are at war, but together against a common enemy. In this battle, health-care workers and researchers fight back against the severe acute respiratory syndrome coronavirus 2 (SARS-CoV-2) threat from the clinic and the laboratory. Behavioural and therapeutic strategies are fundamental, as we highlighted in our last editorial, but they must be coupled with the long-term goal of a preventative vaccine.

Researchers are working to orchestrate an unprecedented global effort to find a vaccine against COVID-19 in record time. The Coalition for Epidemic Preparedness Innovations (CEPI) was established 3 years ago, with the aim of ensuring the world is prepared to deal with new infectious diseases. CEPI is leading efforts to finance and coordinate research on a vaccine for COVID-19 by launching a call for proposals in early February. Several companies and academic institutions working on vaccine candidates answered. CEPI chose eight of them, including Moderna (MA, USA), which, together with US National Institute of Allergy and Infectious Disease, launched the first human trial on a SARS-CoV-2 vaccine on March 16.

The study consists of a safety and immunogenicity phase 1 clinical trial to test mRNA-1273, a novel lipid nanoparticle-encapsulated mRNA-based vaccine that encodes for a full-length, prefusion stabilised spike (S) protein of SARS-CoV-2, in 45 healthy adults. Enrolment begun in Seattle (WA, USA), with Emory University in Atlanta (GA, USA) also recruiting healthy volunteers. On April 14, Moderna stated that the trial is on track and has started enrolling patients to receive the highest dose of the vaccine; there is hope that a phase 2 trial could commence in Spring or early Summer of 2020. The speed of these developments–63 days from sequence selection for mRNA-1273 to the beginning of a human trial for a vaccine candidate– is impressive, owing to both the relentless work of the scientists and the unprecedented demand of the circumstances.

Given the genetic similarity of the two coronaviruses, earlier vaccine research done by Moderna for Middle East respiratory syndrome coronavirus (MERS-CoV) was useful for the design of mRNA-1273. This similarity granted Moderna a selective advantage. In addition, the candidate from Moderna is an RNA vaccine, developed using a technology that, compared with traditional vaccines methods, is faster, cheaper, and easier to scale-up. Because the vaccine is based on a synthetic RNA molecule that encodes for a single viral protein, it also promises to be safer, as it does not involve the attenuation of live viruses. Finally, in the context of the pandemic, global regulators allowed human trials to run in parallel with animal testing, and so human studies could commence before animal results are available.

Richard Hatchett, CEO of CEPI, has chosen to fund a wide range of partners and vaccine technologies to provide the best chance of developing a vaccine that can stop the spread of COVID-19. Besides Moderna, the other candidates funded by CEPI have been developed by both companies and academic institutions. The selected strategies for this race are diverse, as are the stages of research. Common ground for several candidates is that previous knowledge is built on MERS-CoV and severe acute respiratory syndrome coronavirus (SARS-CoV), but this is not the only element in CEPI's strategy. We can see a clear interest in new technologies: while Moderna and CureVac (Tübingen, Germany) are developing an mRNA-based vaccine, Novavax (MD, USA) is using recombinant protein nanoparticle technology to deliver antigens derived from the viral S protein. Other recombinant vaccine approaches have also been considered. Researchers at the University of Hong Kong (Pok Fu Lam, Hong Kong) are using a weakened version of influenza virus that has been altered to express the surface protein of the SARS-CoV-2 virus, and the consortium led by Institut Pasteur (Paris, France) is adopting a measles vaccine as a vector.

The University of Queensland (QLD, Australia) is leveraging on its S-spike vaccine. The candidate has been developed via molecular clamp technology, which uses a lab-created polypeptide to pin the spike protein in its tortile position so that the body's immune system can target it before the virus has a chance to activate. The INO-4800 DNA vaccine, developed by Inovio Pharmaceuticals (PA, USA) has been given permission to do a phase 1 clinical trial in 40 volunteers after showing promising results in animals, and the first dosing was delivered on April 6. The University of Oxford (Oxford, UK) has been selected for its ChAdOx1 vectored vaccine, ChAdOx1 nCoV-19, which is based on an adenoviral vaccine vector already tested and funded by CEPI for other pathogens, including MERS-CoV. After doing animal studies in early March, researchers began recruiting 510 human participants for phase 1 and phase 2 trials on March 27.

Outside of CEPI's funded initiatives, researchers are working to develop translationally relevant candidates and solutions, increasing the odds of finding successful vaccines. Shenzhen Geno-Immune Medical Institute (Guangdong, China) is testing two cellular candidates in phase 1 trials of 100 participants each. Both vaccines use lentiviral vector systems to modify cells to express viral genes and activate T-cells; Covid-19/aAPC vaccine is based on modified artificial antigen presenting cells, whereas the second candidate, LV-SMENP-DC, modifies dendritic cells. CanSino Biologics (Tianjin, China) initiated a phase 1 safety trial on March 18, recruiting 108 participants in Wuhan (China) to test a recombinant adenovirus vaccine candidate, Ad5-nCoV. On April 12, they moved to a phase 2 trial, that will enrol 500 participants.

Several lines of preclinical research are also progressing quickly. Andrea Gambotto and colleagues from the University of Pittsburgh School of Medicine (PA, USA) published a preclinical study in *EBioMedicine* on April 2, showing promising results on animals for the PittCoVacc candidate, built using lab-made pieces of viral protein to build immunity. The study also tested a novel delivering method, a microneedle array with biodegradable needles that deliver the spike protein pieces into the skin, to increase potency and scalability.

The race for a vaccine moves fast, as the need for a solution is evident, but we cannot forget that safety is of the highest importance. Previous work on SARS-CoV and MERS-CoV has contributed to the rapidity of design and development of candidates, whose common goal is to elicit polyclonal antibody responses against the spike protein of SARS-CoV-2 to neutralise viral infection. But reasons for concern have arisen too. In vitro and few in vivo studies on SARS-CoV and MERS-CoV have suggested that antibodies against the virus could cause immune-enhanced disease, either by enhancing infection into target cells, or by increasing inflammation and severity of pulmonary disease. This issue raises the possibility that similar events might occur with SARS-CoV-2 infection. Eng Eong Ooi and colleagues from Duke-NUS Medical School (Singapore) describe in a review in Press at *EBioMedicine* the potential effect of such risk, and the importance of adopting strategies for mitigating the risks right at the outset while developing vaccines or therapeutic antibodies.

While vaccine development research continues, questions are already arising on the next steps and challenges, concerning the manufacturing, distribution, and widespread accessibility of a possible vaccine. Some strategies are already being considered: Sandy Douglas at the University of Oxford, for example, is leading the ChAdOx1 nCoV-19 vaccine manufacturing scale-up project. Working immediately on large-scale production could accelerate the availability of a high-quality and safe vaccine when the right candidate is there.

Of course, once an effective vaccine is available, it will be of the upmost importance to provide affordable and accessible protection from COVID-19 for all who need it. Right now, we celebrate the efforts of scientists, doctors, and individuals working around the clock to find a solution to this pandemic.

*EBioMedicine*

